# Transactions of the Central States Dental Association

**Published:** 1867-02

**Authors:** W. H. Shadoan


### TRANSACTIONS OF THE CENTRAL STATES DENTAL ASSOCIATION.

The Fourth Annual Meeting of the Central States Dental Association, was held in the City of Louisville, Ky., on Wednesday, Thursday, and Friday, December the 26, 27, and 28, 1866.

### FIRST DAY, MORNING SESSION.

The Association met at the Lecture Room of the Kentucky School of Medicine Buildings, at 10 o'clock, A. M. There not being a quorum present, the Society adjourned to meet at the office of Dr. Redman at 2½ o'clock, P.M.

### FIRST DAY, AFTERNOON SESSION, 2½ O'CLOCK, P.M.

The roll was called, and the following members answered to their names: Drs. S. Driggs, W. G. Redman, J. H. Bedford, W. H. Shadoan, E. W. Mason, W. D. Stone, J. F. Canine, G. W. Jones, J. A. McClelland, G. W. Fields, W. H. Goddard, and at a later period, R. H. Wilson, W. F. Morrell, E. Q. Naghel, G. B. Fittz, H. S. Saunders, C. E. Dunn, and J. F. Lampkin.

The minutes of the previous meeting were read.

On motion, Dr. Goddard was elected to fill the vacancy in Executive Committee.

The name of Dr. Francis Peabody was presented for membership, and referred to the Committee on Membership.

Dr. Goddard, one of the Committee on Membership, was excused at his own request from acting in this case, and Dr. Mason appointed in his stead.

The Executive Committee, whose duty it is to procure a place for holding the meetings of this association, made the following report:

The Executive Committee would report that they had secured the room now occupied by Mr. Myers, in the Kentucky School of Medicine, at an expense of four dollars per day, and one dollar additional for night meetings. W. H.

Shadoan, Chairman. On motion, the report was received and adopted.

On motion, That this Association hold their present sittings in the offices of the Dentists of the City. Adopted.

Dr. Shadoan announced the death of Dr. James L. Nourse, a member of this Association, whereupon the following committee was appointed to draft suitable resolutions as the sense of this Association. E. W. Mason, W. H. Shadoan, W. H. Goddard, committee.

On motion, It was voted that this Society hold three sessions each day. To convene at 9 o'clock, A. M., and at 2½, and 7 o'clock, P. M.

On motion, adjourned to meet at the office of Dr. Bedford at 7 o'clock, P. M.

FIRST DAY, EVENING SESSION, 7 O'CLOCK.

Minutes read and approved.

Dr. Driggs, the President, in the Chair.

The Executive Committee would beg leave to make the following report on subjects for discussion during this meeting:

1. Decay in Children's Teeth—Cause and Prevention.
2. Decay in Adult's Teeth—Cause and Prevention.
3. Irregularities—Causes and Prevention.
4. Miscellaneous.

On motion, the Association will have clinic operation at 10 and 3 o'clock each day.

The first subject, Decay in Children's Teeth, Causes and Prevention, was then taken up and discussed.

The Committee on Membership reported the name of Dr. Peabody as a suitable person to become a member of this Society.

Whereupon a ballot was had, resulting unanimously in his favor.

Adjourned to meet at the office of Dr. Canine at 9 A. M. on to-morrow.

SECOND DAY, FORENOON SESSION, 9½ O'CLOCK.

Minutes read, corrected and adopted.

After some desultory remarks, the President announced the time of selecting the place of holding the semi-annual meeting.

On motion of Dr. Lampkin, Lexington, Ky., was selected.

On motion the first subject, decay in children's teeth, causes and prevention, was then taken up and finished.

The second subject, decay in adult teeth, was then taken up and discussed at considerable length.

Adjourned to meet at the office of Dr. Goddard, at 2½ P. M.

SECOND DAY, AFTERNOON SESSION, 2½ O'CLOCK.

Minutes read and approved.

A letter was read from Dr. Rogers, of Shelbyville, expressing regrets at not being able to attend the meeting, on account of sickness, &c.

The hour for holding the election of officers having arrived, on motion, it was postponed until after the clinic.

The members then indulged in witnessing a clinic operation by Dr. McClelland, which was watched by all present with deep interest.

Dr. G. W. Acree, of Memphis, Tenn., was then introduced, who presented the following certificate from the Memphis Dental Association, which was read and referred to the Committee on Membership. The certificate reads as follows:

MEMPHIS, TENN., *Dec.* 5, 1866.

This certifies that at a regular meeting of the Memphis Dental Association, held on the eve of the 4th inst., Dr. G. W. Acree was elected a Delegate to represent this Society in the Convention of Dentists, to assemble in the city of Louisville, on the 26th inst.

Attest:

L. W. FLETCHER, Sec'y.

The Committee reported upon the certificate, and recommended Dr. Acree as a suitable person to become a member.

The clinic being over, the Society went into an election of officers for the ensuing year, which resulted as follows:

President—Dr. W. G. Redman, of Louisville;
1st Vice President—Dr. E. W. Mason, "
2d Vice President—Dr. W. H. Goddard, "
3d Vice President—Dr. J. A. McClelland, "
Secretary—Dr. W. H. Shadoan, "
Cor. Secretary—Dr. J. F. Canine, "
Treasurer—Dr. R. H. Wilson, "

Drs. Goddard and Stone were appointed a committee to conduct officers to their place.

A ballot was then had for Dr. Acree for membership, resulting in his favor.

Adjourned to meet at Dr. Shadoan's office, at 7 o'clock P. M.

SECOND DAY, EVENING SESSION, 7½ O'CLOCK.

President Redman in the Chair. Minutes read and approved.

The President appointed the Standing Committees:

Executive Committee—Drs. J. Taft, E. W. Mason and S. Driggs.

Committee on Membership—Drs. Goddard, McClelland and Acree.

The second subject was then taken up and further discussed. by Drs. Stone, Taft, McClelland, Canine, and others.

On motion, Exposed Pulps were discussed, several members taking part.

On motion, the subject was closed.

The resolution fixing Lexington as the place of holding the Semi-Annual Meeting in April next, was reconsidered. After some discussion, of an explanatory character, Memphis, Tenn., was selected.

Adjourned to meet at the office of Dr. McClelland at 9 o'clock, A. M., on to-morrow.

THIRD DAY, FORENOON SESSION 9 O'CLOCK.

President Redman in the Chair. Minutes read and approved.

The 3d subject, Irregularity of the Teeth, was then taken up and discussed.

On motion, the subject was discontinued and the claims of the Goodyear Dental Vulcanite Company was considered.

Dr. McClelland read an Essay on Dentrifices. A vote of thanks was tendered the Dr. for his essay, and a copy requested for publication.

The meeting adjourned to meet at the office of Dr. Wilson, at 2 o clock, P. M.

THIRD DAY, AFTERNOON SESSION, 3 O'CLOCK.

Dr. Goddard in the Chair; Dr. Field, Sec'y. *Pro. Tem.*

On motion of Dr. Acree, a Committee was appointed to draft resolutions on the death of Dr. A. M. Leslie, of St. Louis, who died in Memphis in November last.

The Committee consisted of Drs. Acree, McClelland and Naghel.

The Committee appointed to draft a petition praying for suitable protection against Dental Quackery, which is as follows, viz :

*To the Honorable the Legislature of the State of Kentucky:*

Your petitioners would respectfully represent that Dental Surgery being a specialty of the healing art, requires for its proper performance a knowledge of Anatomy, Physiology, Pathology, Therapeutics, Chemistry, and the theory and practice of Surgical and Mechanical Dentistry. The acquisition of a knowledge of these different branches requires at least two years of close application to study, with competent instructors.

Not until we are enlightened upon a subject can we appreciate the importance that attaches to it, and as the public have no means of judging between the competent and incompetent Dentist, they should, in justice, have some guaranty of qualification.

While the older and leading practitioners of Dental Surgery acknowledge THEIR NEED OF MORE LIGHT the people of this Commonwealth are being grossly imposed upon by the MEREST PRETENDERS to Dental science, without possessing a knowledge of the first principles requisite to its successful practice; hence much suffering, discomfort and ill-health results that might and should be averted.

Your petitioners, therefore, respectively pray your honorable body to protect the citizens of the Commonwealth of Kentucky from injury by incompetent Dental practitioners, by such enactments as in your wisdom you may deem sufficient.

S. Driggs, W. H. Goddard, W. G. Redman, J. A. McClelland, W. H. Shadoan, E. W. Mason, *Committee.*

W. M. Rogers, J. W. Baxter, A. S. Talbert, J. F. Canine, G. S. Jones, H. Baldwin, Samuel Griffith, F. Peabody, W. D. Stone, R. C. Morgan, H. McCullum, J. H. Bedford, J. G. Van Marter.

The Committee then reported a Bill which was adopted and is as follows, viz:

*A Bill to Regulate the Practice of Dentistry in the State of Kentucky.*

SECTION 1. Be it enacted by the General Assembly of the State of Kentucky, That after the first day of January, 1868, it shall be unlawful for any person to practice Dentistry in the State of Kentucky, unless such person has received a diploma from a faculty of a Dental College duly incorporated under the laws of this or any other State of the United States, or a certificate of qualification from the State Board of examiners hereinafter specified.

SEC. 2. Said board of examiners shall consist of three practitioners of Dentistry, possessing the evidence of qualification contemplated in this Act. They shall be appointed, and vacancies filled by the Governor, by and with the consent of the Senate.

SEC. 3. The board of examiners shall serve for a term of three years, and until their successors are installed, except the members of the first board, one of whom shall serve for one year, one for two years, and one for three years.

SEC. 4. The board of examiners shall meet at least once a year for the purpose of examining applicants; after having giving at least sixty days notice of such meeting in some newspaper of general circulation throughout the State. They shall also have power to make such arrangements as shall be necessary for the prompt and efficient performance of their work as such examiners.

SEC. 5. Any one member of the board of examiners, on a satisfactory examination of applicant, shall grant him permission to practice until the regular session of the board.

SEC. 6. Each applicant shall, on receiving a certificate, from the board, pay into the treasury the sum of 10 dollars, which fund shall be used by the board for the benefit of the Dental profession in the State.

SEC. 7. Any person who shall practice Dentistry without having complied with the requisitions of this Act, shall, for each offence, be deemed guilty of a misdemeanor, and upon conviction thereof, shall be fined not less than ten dollars, nor more than fifty dollars; provided that nothing in this Act shall be construed to prevent physicians and surgeons from extracting teeth.

SEC. 8. All prosecutions under this Act shall be by indictment before the circuit court in the county where the offence shall have been committed, and all fines imposed and collected under this Act, shall be paid into the treasury of the county where such conviction shall take place, for the use of the common schools within such county.

SEC. 9. This Act shall take effect and be in force from and after its passage.

On motion, the Committee was discharged, and the following gentlemen appointed to carry into execution the design of the petition. Drs. Driggs, Mason, Shadoan, McClelland and Goddard.

The Treasurer made his Report which is as follows:—
[Report did not come to hand.—EDITOR.]

Drs. Goddard, Naghel and Jones were appointed to audit the report; who, after a few minutes absence, reported that they had examined his report and found it correct.

Signed Goddard, Naghel and Jones.

The Committee appointed to draft Resolutions expressive of the sense of this Society on the death of Dr. A. M. Leslie, made the following report, which was adopted.

On the announcement of the death, in Memphis, of Dr. A. M. Leslie of St. Louis, the Central States' Dental Association, now in session in Louisville,

*Resolved*, That in the death of Dr. Leslie, the Dental profession has lost a worthy co-laborer. Though not actively in practice for the last few years, he ever maintained a lively interest in the progress of Dental science, and was always foremost in furnishing such material devices as the profession most required.

*Resolved*, That the bereaved friends and family have our warmest sympathy.

G. W. ACREE,
J. A. MCCLELLAND, } *Committee.*
E. Q. NAGHEL,

Adjourned to meet at the office of Dr. Mason, at 7 o'clock, P. M.

THIRD DAY, EVENING SESSION.

Minutes read and approved.

Miscellaneous subjects taken up. The question of the demands of the Goodyear Dental Vulcanite Company. After nearly all expressing their disapproval of the course of the company, the following resolution was offered by the Secretary, which, after some slight amendments, was adopted:

*Resolved*, By the Central States Dental Association, that we feel it to be the duty of all Dentists to act in conjunction with others in resisting the demands of the Goodyear Dental Vulcanite Company, believing, as we do, that their terms are unreasonable and unjust, and ought to be resisted.

The Committee appointed to draft suitable resolutions on the death of one of our members, Dr. Nourse, made the following report, which was adopted:

Resolutions of respect on the death of Dr. J. L. Nourse:

WHEREAS, It has pleased an allwise and inscrutable Providence to remove from our midst and from this Association by death, our much esteemed friend and brother, Dr. J. L. Nourse, of Cloverport, Ky., in August last; therefore

1. *Resolved*, That in the death of Dr. Nourse, that we, as a body, and those of us who were more intimately acquainted with him, have lost a valuable member, and true friend.

2. *Resolved*, That in the death of Dr. Nourse, society has sustained an irreparable loss, and the Dental profession one of its best members.

3. *Resolved*, That of Dr. Nourse we will cherish the fondest recollections, and the most profound respect.

4. *Resolved*, That we deeply sympathize with his friends and his aged parent in particular, in their bereavement.

5. *Resolved*, That these resolutions be published with the minutes of this Association in the DENTAL REGISTER.

E. W. MASON,<br>W. H. SHADOAN. } *Committee.*

Notice is hereby given that at the next Annual Meeting, a resolution will be offered in regard to the propriety of changing the time of holding the meetings of this Society.

On motion of Dr. Driggs, a vote of thanks was tendered

Dr. Field, the retiring secretary, for his services and for his report of discussions at the last meeting.

Resolved that a vote of thanks be tendered all the retiring officers, and Dr. Driggs in particular, for the able manner in which he presided over the Society.

Dr. Driggs then made some excellent remarks in reference to the advance that Dentists should take as professional men; that this Society should elevate the standard, and live up to the highest advance.

Adjourned to meet in the city of Memphis, Tenn., on Tuesday, April 9, at 10 o'clock A. M., 1867.

W. H. SHADOAN,
*Secretary.*

